# The monounsaturated fatty acid oleate is the major physiological toxic free fatty acid for human beta cells

**DOI:** 10.1038/s41387-017-0005-x

**Published:** 2017-12-21

**Authors:** T Plötz, B Krümmel, A Laporte, A Pingitore, SJ Persaud, A Jörns, M Elsner, I Mehmeti, S Lenzen

**Affiliations:** 1 Institute of Clinical Biochemistry, Hannover Medical School, Hannover, Germany; 2 Institute of Experimental Diabetes Research, Hannover Medical School, Hannover, Germany; 3 Institute of Medical Biochemistry and Molecular Biology, University Medicine Greifswald, Greifswald, Germany; 4 Division of Diabetes and Nutritional Sciences, Faculty of Life Sciences and Medicine, King’s College London, London, UK

## Abstract

Free fatty acids (FFAs) can cause glucose intolerance and diabetes. Lipotoxicity to the pancreatic beta cells is considered to be a major underlying cause for this phenomenon. The aim of this study was to analyse the toxicity profile of FFAs in the human EndoC-βH1 beta-cell line and to compare the results with isolated rat and human islets with special reference to the physiologically most prevalent FFAs palmitic acid (PA) and oleic acid (OA). Toxicity after a 2-day incubation with the different FFAs was analysed by the caspase-3 assay and confirmed by the propidium iodide and annexin V staining tests. The long-chain saturated PA (C16:0) and the monounsaturated OA (C18:1) were both toxic to human EndoC-βH1 beta cells and pseudoislets, as well as to rat islets, and, as confirmed in a pilot experiment, also to human islets. Furthermore, OA provided no protection against the toxicity of PA. Likewise, elaidic acid (EA, the *trans* isomer of OA; *trans*-OA) was significantly toxic, in contrast to the non-metabolisable analogues methylated PA (MePA) and methylated OA (MeOA). Fatty acids with a chain length  < C16 were not toxic in EndoC-βH1 beta cells. Caspase-3 was also activated by linoleic acid (LA)(C18:2) but not by γ-linolenic acid (γ-LNA)(C18:3). Overall, only long-chain FFAs with chain lengths  > C14, which generate hydrogen peroxide in the peroxisomal beta-oxidation, were toxic. This conclusion is also supported by the toxicity of the branched-chain FFA pristanic acid, which is exclusively metabolised in the peroxisomal beta-oxidation. The lack of a protective effect of the monounsaturated fatty acid OA has important consequences for a beta-cell protective lipid composition of a diet. A cardioprotective diet with a high OA content does not fulfil this requirement.

## Introduction

There is a firm belief in the general population and in the medical profession that a Mediterranean diet reduces the incidence of cardiovascular disease^1,2^. This has been thought to be the explanation for the decreasing rate of coronary heart disease mortality in many populations^1,2^. This conviction originates from early epidemiological studies^3^, a view that has been maintained over many decades^4,5^. The high content of the monounsaturated fatty acid (MUFA) oleic acid (OA) in olive oil is typically thought to be crucially responsible for this cardioprotective effect when compared with diets containing oils with a dominant content of saturated fatty acids (SFAs)^1,4,6^. This concept has had considerable influence on public health policy recommendations regarding fat content and composition of the daily diet.

Central to this concept is the promotion of the consumption of MUFAs as a more healthy substitute for SFAs in the daily diet by the American Heart Association, as well as the US Food and Drug Administration and by major learned societies (https://www.guideline.gov/summaries/summary/3127). Diets with high MUFA contents have also been considered favourable in counteracting diabetes development^7^, although evidence has never been unequivocal^8^.

A critical review of the International Nutrition Guidelines for Diabetes comprising an evaluation of the recommendations of six learned international scientific societies by Katsilambros et al. for the Diabetes and Nutrition Study Group of the European Association for the Study of Diabetes (http://www.dnsg-easd.org/info/general/Document/get/18/documentId/KATSILAMBROS%20et%20al.pdf) revealed a unified recommendation for a low intake of SFAs and a minimisation of trans fatty acid intake along with a clear preference for MUFA consumption. This recommendation has been reiterated recently in the Guidelines of the European Society of Cardiology (ESC), which was developed in collaboration with the European Association for the Study of Diabetes (EASD) (https://www.escardio.org/static_file/Escardio/Guidelines/publications/DIABETWeb_EM_Diabetes_2013.pdf).

Nevertheless, doubts have been developing during recent years regarding the validity of this concept^1,6,9^, because a number of basic studies failed to confirm the protective effect of MUFAs against SFA-promoted atherogenesis^1,6^. In particular, a cardioprotective effect of OA, representing the principal MUFA component of olive oil, could not be documented^1,6^. Similar doubts have been expressed with respect to an antidiabetic effect of MUFAs^8^.

An antilipotoxic effect of OA and other MUFAs has been documented in many studies on insulin-secreting cell lines of rodent origin^10^. Whether this also applies to human pancreatic beta-cell lines, however, is unknown.

In the present study, we address this question, making use of the new human EndoC-βH1 beta-cell line and for comparison of isolated rat islets, as well as of isolated human islets in a pilot analysis. We show in this study that the MUFA OA is not protective against PA-mediated toxicity to human beta cells. Rather, this main dietary MUFA turned out to be as toxic as PA, the main dietary SFA in humans^11,12^.

This observation is likely to have significant impact on future recommendations for a healthy diet composition with respect to type 2 diabetes (T2DM) prevention, with the aim of counteracting the worldwide rapidly increasing T2DM incidence (http://www.diabetesatlas.org/resources/2017-atlas.html).

## Materials and methods

### Chemicals and media

Free fatty acids (FFAs) were purchased from Sigma-Aldrich (Munich, Germany). Cell culture medium (Dulbecco’s modified Eagle’s medium (DMEM); Life Technologies, Darmstadt, Germany) was supplemented with 5.5 mM glucose, 2% bovine serum albumin (BSA; Serologicals Proteins Inc, Kankakee, IL, USA), 1% penicillin/streptomycin (Biochrom, Berlin, Germany), 50 µM 2-mercaptoethanol, 10 mM nicotinamide, 5.5 µg/ml transferrin, 6.7 ng/ml sodium selenite (all from Sigma-Aldrich). FFA free BSA was from Serva (Heidelberg, Germany).

### Human EndoC-βH1 beta-cell line as well as rat and human islet culture

EndoC-βH1 beta cells (ENDOCELLS SARL, Paris, France) were cultured^13^ and pseudoislets (PIs) were generated and cultured under the same conditions as described previously^13^. Rat pancreatic islets were isolated from six different 250–300 g adult male Lewis rats by collagenase digestion, separated by Ficoll gradient and handpicked under a stereo microscope^10^. Human islets were isolated at the King’s College Hospital Islet Transplantation Unit, with appropriate ethical approval (LREC 01-082)^14^. The human islets used in this study were isolated from the pancreas of a 49-year-old non-diabetic male donor (body mass index (BMI): 30.3 kg/m^2^; blood glucose: 5.3 mM). Islets were maintained in Connaught Medical Research Laboratories (CMRL) medium supplemented with 2% human albumin, 4 mM glutamine, 2 mM HEPES (pH 7.2–7.4) and 10 mM nicotinamide at 37°C, 5% CO_2_ prior to functional analyses. FFA stock solutions (50 mM) were prepared in 90% ethanol. Incubation medium in the final concentrations was prepared using DMEM medium with fatty acid-free BSA^10^.

### Toxicity analyses

Groups of 30 000 human EndoC-βH1 beta cells, 50 human EndoC-βH1 PIs, 10 rat islets or 5 human islets, cultured on 96-well plates, were incubated for 2 days with the various FFAs. Caspase-3 activity was quantified by the CaspaseGlo-3/7 kit (Promega, Mannheim, Germany). Propidium iodide (5 µg/ml) (Sigma-Aldrich) staining was quantified after a 15-min incubation at 37°C with the dye by flow cytometry in the FL-3 channel as described before^13^. Annexin V staining was quantified after a 15-min incubation at 37°C with the dye by fluorescence microscopy using the Annexin V-FITC apoptosis kit (Thermo Fisher Scientific, Waltham, MA, USA). Apoptotic EndoC-βH1 beta-cell nuclei were identified by electron microscopy as described^10^.

### Statistical analysis

Data are expressed as means ± SEM. Statistical analyses were performed using analysis of variance plus Dunnett’s multiple comparison test and Student’s *t*-test, unless stated otherwise (Graphpad, San Diego, CA, USA).

## Results

**Fig. 1:**
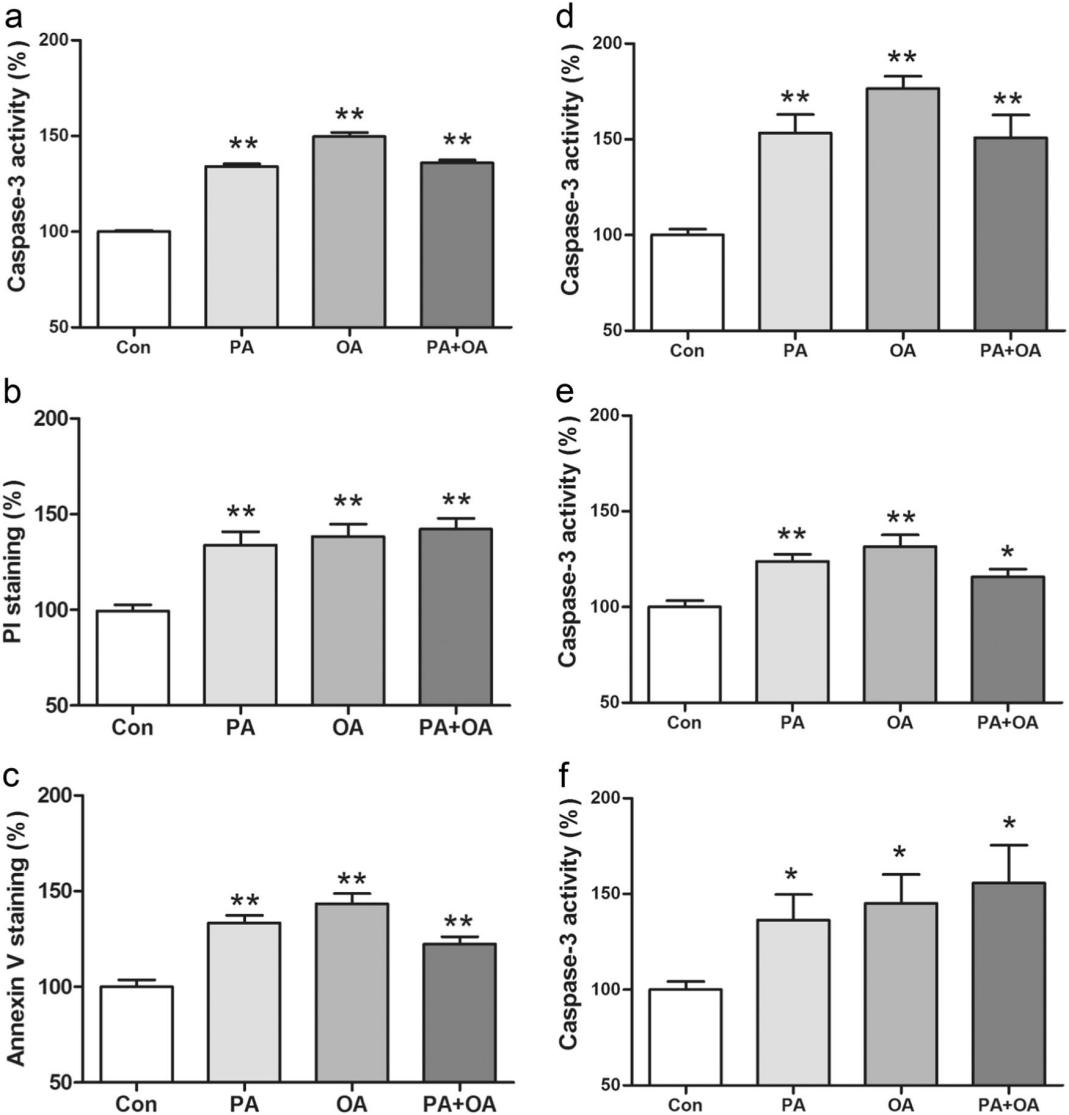
Toxicities of PA, OA or the combination in human EndoC-βH1 beta cells by three different apoptosis tests **a**-**c**, and in human EndoC-βH1 PIs **d**, isolated rat islets **e**, and isolated human islets **f**. Cells and islets were incubated for 2 days with PA (500 μM), OA (500 μM) or a combination of both (500 μM each). Thereafter, toxicity was determined by measurements of caspase-3 activity **a**, **d**-**f**, propidium iodide (PI) staining **b** or annexin V staining **c**. Data are means ± SEM of 5–7 experiments from independent preparations in the EndoC-βH1 beta-cell experiments **a**-**e**. In the case of the rat islet studies, islets were isolated from six different animals **d** and in the case of the human islet studies from a single human donor **f**. *p < 0.05, **p < 0.01 compared with untreated control cells.

Caspase-3 was significantly activated in human EndoC-βH1 beta cells (Fig. 1a), human EndoC-βH1 PIs (Fig. 1d), isolated rat pancreatic islets (Fig. 1e) and, in a pilot study, isolated human pancreatic islets (Fig. 1f) after a 2-day treatment with the physiologically most prevalent saturated long-chain FFA PA (C16:0)(500 μM), as well as with the monounsaturated long-chain FFA OA (C18:1)(500 μM). Independent confirmation for apoptotic cell death induced by the two FFAs was obtained in human EndoC-βH1 beta cells by toxicity analyses with the propidium iodide (Fig. 1b) and annexin V (Fig. 1c) staining tests. A combination of both FFAs (500 μM each) was also toxic, but an additive effect was not observed (Figs. 1a-f).

Toxicity was detected with FFAs having a minimum chain length of C16. Shorter FFAs were not toxic to human EndoC-βH1 beta cells. Thus, the medium-chain myristoleic acid (C14:1) (100 ± 3%, *n* = 4) did not increase caspase-3 activity as compared with the control situation (100 ± 2%, *n* = 4).

**Fig. 2:**
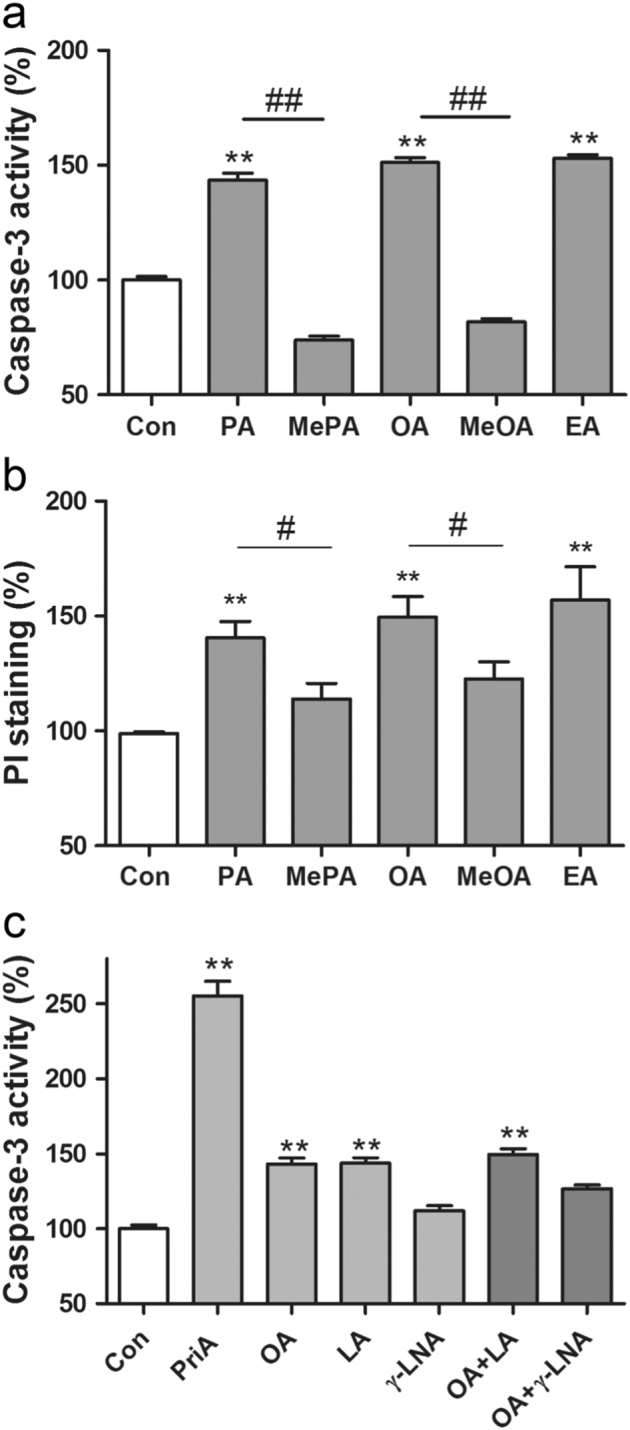
Toxicities of PA, MePA, OA, MeOA and EA (*trans*-OA) **a**-**b** and toxicities of the PUFAs LA (C18:2) and γ-LNA (C18:3) in comparison with the MUFA OA (C18:1) and to PriA in human EndoC-βH1 beta cells and their ability to antagonise the toxic effect of OA (C18:1) **c**. EndoC-βH1 beta cells were incubated for 2 days with PA, MePA, OA, MeOA and EA (*trans*-OA)(all FFAs 500 µM). Thereafter, caspase-3 activity **a** and propidium iodide fluorescence **b** were measured. In addition, EndoC-βH1 beta cells were incubated for 2 days with the polyunsaturated fatty acids (PUFAs) LA (500 µM), γ-LNA (500 µM), and for comparison with the monounsaturated OA (500 µM), as well as the branched-chain PriA (200 µM). Furthermore, combinations of OA with the PUFAs (500 µM each) were incubated. Thereafter, caspase-3 activity was measured **c**. Data are means ± SEM of 5–8 independent experiments. **p < 0.01 compared with untreated control cells; ^#^ *p* < 0.05, ^##^ *p* < 0.01 compared with the corresponding non-methylated fatty acid.

Not only PA and OA (*cis*-OA), but also EA (elaidic acid, the *trans* isomer of OA; *trans*-OA) caused significant toxicity to human EndoC-βH1 beta cells in contrast to the non-metabolisable analogues methylated PA (MePA) and methylated OA (MeOA) (Figs. 2a, b). These results were obtained by the caspase-3 apoptosis assay (Fig. 2a) and confirmed by the propidium iodide staining test (Fig. 2b). Further independent proof for apoptotic cell death caused by PA and OA has been provided by identification of apoptotic EndoC-βH1 beta-cell nuclei by electron microscopy (Supplementary Fig. S1).

Caspase-3 was also significantly activated in human EndoC-βH1 beta cells after a 2-day treatment with the FFA linoleic acid (LA)(C18:2), but not with γ-linolenic acid (γ-LNA)(C18:3). Although LA showed a degree of toxicity similar to OA, γ-LNA was not significantly toxic to human EndoC-βH1 beta cells (Fig. 2c). A significantly lower toxicity could also be documented for OA when combined with γ-LNA (C18:3), but not when combined with LA (C18:2) (Fig. 2c). The branched-chain fatty acid pristanic acid (PriA), which is exclusively metabolised in peroxisomal beta oxidation, was very toxic in these analyses (Fig. 2c).

The results of the present study document that the physiological MUFA OA is at least as toxic as the SFA PA to human beta cells. OA, the physiologically most abundant MUFA, which is even more prevalent in humans than PA^11,12^, cannot protect human beta cells against PA toxicity. And EA, the *trans* isomer of OA showed toxicity comparable to that of *cis*-OA.

As the non-metabolisable methylated analogues MePA and MeOA showed no significant toxicity to human EndoC-βH1 beta cells, the data support the conclusion that metabolism of the fatty acids is an indispensable prerequisite for their toxicity.

## Discussion

Our data indicate that results obtained in previous lipotoxicity studies in rat insulin-producing cell lines cannot be simply extrapolated to primary pancreatic islets as OA, in contrast to PA, was not or only minimally toxic and provided protection against the toxicity of SFAs such as PA in these insulin-producing rat cell lines^10,15–17^. In contrast, we document in the present study by three different apoptosis assays, as well as by electron microscopy the toxicity of OA and the inability of OA to reverse the toxic effect of PA. Beta cells of the permanent human EndoC-βH1 beta-cell line are a good substitute as they mirror the situation in rat, as well as in human primary pancreatic islets. On the other hand, OA in combination with PA did not show an additive toxic effect. Thus, a combined presence of SFAs and MUFAs in the diet, as well as in the physiological lipid composition in the human body is probably the optimum that can be achieved with respect to minimisation of lipotoxicity in the organism. Therefore, an emphasis on raising the proportion of MUFAs such as OA in diets too much and thereby creating an imbalance in favour of the MUFA component, through consumption of excessive amounts of vegetable oils such as olive oil, can be considered as counterproductive for beta-cell protection. Under this perspective, a vegetable oil with plenty of antioxidants but a limited content of OA might be the optimum that can be achieved when selecting a vegetable oil brand preparation, that is, for salad dressings.

PUFAs with more than two double bonds were less toxic and did provide protection against the toxicity of SFAs and MUFAs in human EndoC-βH1 beta cells. The proportion of PUFAs with more than two double bonds in human plasma is only around 3%^11^. So the amount of these PUFAs is insufficient to compensate for the toxicity of saturated and mono- and diunsaturated fatty acids in human beta cells.

Thus, the pancreatic beta-cell cannot be protected against the toxicity of SFAs through increased uptake of MUFAs. Therefore, it may be that the beta cell has to pay the price for the current emphasis on the consumption of a cardioprotective high MUFA diet. It is thus tempting to consider such a diet as a contributory factor to the continuing worldwide increase in the incidence of T2DM during recent decades (http://www.diabetesatlas.org/resources/2015-atlas.html).

From studies in rat insulin-producing cell lines, it is known that hydrogen peroxide is the responsible toxic reactive species in PA-induced beta-cell toxicity^18^. This hydrogen peroxide is preferentially generated in the beta-oxidation in the peroxisomes^18^, which has a preference for saturated and unsaturated fatty acids with chain lengths >14 C-atoms^10^. This is exactly the preference for beta-cell lipotoxicity of long-chain fatty acids in human EndoC-βH1 beta cells that we document in the present study. Further support for the origin of the toxic hydrogen peroxide in the peroxisomal fatty acid metabolism comes from the observation that the branched-chain fatty acid PriA, which exclusively undergoes peroxisomal beta-oxidation^10,19,20^, was also shown to be toxic in the present study. PriA is the crucial reference substrate for peroxisomal beta-oxidation^15^. So it can be concluded that the different fatty acids, which have been found to be beta-cell toxic in the present study, are without exception substrates for the peroxisomal beta-oxidation^15,19–21^. Therefore, the present results support the concept of beta-cell lipotoxicity through peroxisomal stress^15^ that is mediated by hydrogen peroxide generation in the peroxisomal beta oxidation of the beta cells, which, at variance from virtually all other cell types, lack expression of the hydrogen peroxide inactivating enzyme catalase in the peroxisomes^13,22^.

The results obtained in the present study indicate that primary beta cells of rat and human origin, as well as human EndoC-βH1 beta cells differ from rat insulin-producing cell lines, in that these cell lines show no toxicity upon exposure to unsaturated fatty acids^10,15-17^ due to their inability to generate toxic hydrogen peroxide^10^, as they apparently cannot metabolise unsaturated fatty acids in the peroxisomal beta-oxidation.

## Electronic supplementary material

Figure S1
