# Supplementary material for: The monounsaturated fatty acid oleate is the major physiological toxic free fatty acid for human beta cells
Source: Nutr Diabetes. 2017 Dec 21;7(12):305. doi: 10.1038/s41387-017-0005-x (PMC5865546; doi:10.1038/s41387-017-0005-x)
Supplement: Supplementary file 1 [file 41387_2017_5_MOESM1_ESM.docx]

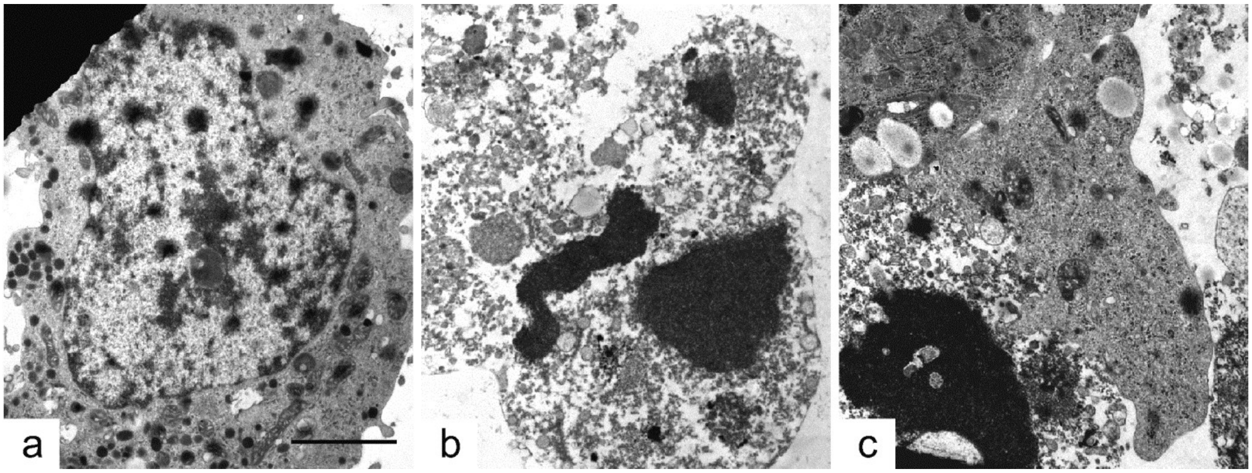
a
b
c
